# Creatine, L-Carnitine, and *ω*3 Polyunsaturated Fatty Acid Supplementation from Healthy to Diseased Skeletal Muscle

**DOI:** 10.1155/2014/613890

**Published:** 2014-08-28

**Authors:** Giuseppe D'Antona, Seyed Mohammad Nabavi, Piero Micheletti, Arianna Di Lorenzo, Roberto Aquilani, Enzo Nisoli, Mariangela Rondanelli, Maria Daglia

**Affiliations:** ^1^Department of Molecular Medicine and Laboratory for Motor Activities in Rare Diseases (LUSAMMR), University of Pavia, Via Forlanini 6, 27100 Pavia, Italy; ^2^Applied Biotechnology Research Center, Baqiyatallah University of Medical Sciences, P.O. Box 19395-5487, Tehran, Iran; ^3^Department of Experimental and Forensic Medicine, University of Pavia, Via Forlanini 2, 27100 Pavia, Italy; ^4^Department of Drug Sciences, Medicinal Chemistry and Pharmaceutical Technology Section, University of Pavia, Via Taramelli 12, 27100 Pavia, Italy; ^5^Maugeri Foundation IRCCS, Montescano Scientific Institute, Via Per Montescano 31, 27040 Montescano, Italy; ^6^Center for Study and Research on Obesity, Department of Medical Biotechnology and Translational Medicine, University of Milan, Via Vanvitelli 32, 20129 Milan, Italy; ^7^Human Nutrition Section, Health Sciences Department, University of Pavia, Azienda di Servizi alla Persona, Via Emilia 12, 27100 Pavia, Italy

## Abstract

Myopathies are chronic degenerative pathologies that induce the deterioration of the structure and function of skeletal muscle. So far a definitive therapy has not yet been developed and the main aim of myopathy treatment is to slow the progression of the disease. Current nonpharmacological therapies include rehabilitation, ventilator assistance, and nutritional supplements, all of which aim to delay the onset of the disease and relieve its symptoms. Besides an adequate diet, nutritional supplements could play an important role in the treatment of myopathic patients. Here we review the most recent *in vitro* and *in vivo* studies investigating the role supplementation with creatine, L-carnitine, and *ω*3 PUFAs plays in myopathy treatment. Our results suggest that these dietary supplements could have beneficial effects; nevertheless continued studies are required before they could be recommended as a routine treatment in muscle diseases.

## 1. Introduction

The role of many foods/nutrients in maintaining good health and prolonging human lifespan has been clearly demonstrated over the past three decades. Particularly important are not only plant foodstuffs (i.e., fruits, vegetables, and legumes), but also animal foods (i.e., fish) and lipids (flaxseed and olive oils), that have been shown to have protective effects against several chronic pathologies such as age-related diseases, including cardiovascular [1], neurodegenerative [2], and inflammatory diseases [3], diabetes [4], and myopathies. Myopathies can be classified as either hereditary or acquired. Congenital myopathies are a group of inherited neuromuscular disorders whose main pathological features are distinctive and specific morphologic abnormalities in skeletal muscle. In contrast, differently acquired myopathies are caused by muscle fatigue, electrolyte imbalance, and dehydration or are induced by immune disorders that cause inflammation and pain [5]. In these pathologies the skeletal muscle is the fundamental target.

The deterioration of skeletal muscle structure and function leads to clinically relevant complaints, including progressive strength loss, fatigue, myalgias, and cramps. Important progress has been made in the comprehension of the molecular mechanisms underlying muscle myopathies. However, the treatment of muscle diseases is mainly symptom-oriented and includes physical therapy, physical exercise, orthopaedic corrections, artificial ventilation in cases of respiratory insufficiency, and pharmacologic interventions (i.e., corticosteroids). Considering the lack of therapies for myopathies, the idea that nutritional supplements might have beneficial effects in myopathy treatment is experiencing renewed interest. Conclusions about how beneficial nutritional supplements are for myopathy treatment are complicated by a lack of unequivocal results and flaws in the choice of supplements.

On the basis of their physiological roles in muscle biochemistry and bioenergetics, the nutrients creatine, L-carnitine, and *ω*3 polyunsaturated fatty acids (*ω*3 PUFAs) have been the focus of research aimed at verifying their safety and efficacy in treatment of a number of muscle diseases. Our aim here is to review the results of the most recent* in vitro* and* in vivo* research on the supplementation of creatine, L-carnitine, and *ω*3 PUFAs.

## 2. Creatine

### 2.1. The Nutritional Biochemistry of Creatine

**Figure 1:**
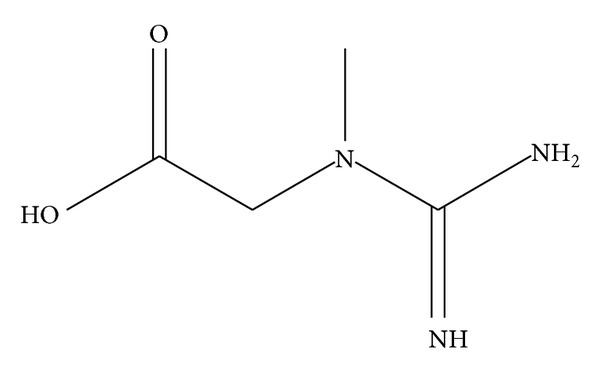
Chemical structure of creatine.

Creatine (*N*-aminoiminomethyl-*N*-methylglycine, Figure 1) is an endogenous guanidine compound, which is synthesized by the kidneys, pancreas, and liver, starting from three amino acids: (1) methionine, which provides the methyl group through a transmethylation reaction, (2) glycine, which provides the acetic group and the nitrogen atom, and (3) arginine, which provides the amide group. Once produced, creatine is released into the bloodstream and then mainly captured by the cardiac and skeletal muscle and brain. To carry out its physiological role, creatine is converted to phosphocreatine by creatine kinase. The donor of the phosphate group is adenosine triphosphate (ATP), which is converted into adenosine diphosphate (ADP). Phosphocreatineis ahigh-energy reserve, availablefor theconversionof ADP to ATP, that is, essential during periods of high energy demand such as intense physical activity. Creatine kinase catalyzes the reversible transfer of the* N*-phosphoryl group from phosphorylcreatine to ADP to regenerate ATP. In this way creatine levels are restored [6]. In a 70 kg man, the total body creatine content is about 120 g, with a turnover of about 2 g/d, corresponding to 1.6% of total body creatine. On average, 50% of an individual's daily requirement of creatine is ingested from foods (approximately 1 g/d), while the remaining 50% is synthesized endogenously. Exogenous dietary sources of creatine include meat and fish (the concentration of creatine ranges between 4 and 5 g/kg of meat and 4 and 10 gr/kg of fish), and other animal products.

To date, the effects of creatine supplementation on muscle growth and muscle performance have been documented in more than 400 publications. Scientific evidence suggests that creatine supplementation (with a considered safe loading dose of 4 g of creatine monohydrate, 4 times per day), is an effective strategy to increase muscle creatine content by up to 10–40%, in less than a week [7]. This supplementation induces the increase of anaerobic performance, training volume, and capacity of human muscle to perform work during alternating intensity contraction [8].

Moreover, creatine also plays a pivotal role in brain energy homeostasis. It has been shown that in some psychiatric disorders, such as depression, the levels of creatine are low [9]. Therefore, recent studies have investigated the effect of creatine monohydrate supplementation in psychiatric patients suffering from posttraumatic stress disorders and depression. Creatine supplementation seems to also show neuroprotective effects in some neurodegenerative pathologies such as Alzheimer's and Parkinson's diseases. Recent evidence suggests that creatine could play important roles in the dysfunction of mitochondrial metabolism, which is recognized as a central causal factor in the pathogenesis of neurodegenerative disorders [10]. In a phase-II clinical trial, creatine monohydrate showed a delay in the progression of Parkinson's disease by 50%, compared to controls that received placebo. Then, in a subsequent followup study carried out 18 months later, creatine continued to show efficacy as a neuroprotective agent. The authors concluded that for its safety, tolerability, activity, and cost, creatine has many advantages in comparison with other drugs or food supplements potentially useful for Parkinson's disease [11, 12].

### 2.2. The Effects of Creatine Supplementation on Muscle Diseases

Creatine monohydrate supplementation, at dose of 0.3 g/kg/d for six days or 0.04 g/kg/d for 30 days [13], induces an increase in total creatine and phosphocreatine concentrations in skeletal muscle [13, 14], the magnitude of accumulation being inversely related to available endogenous stores [14]. It is accepted that the dosing regimen that significantly increase the intracellular phosphocreatine is a loading phase of approximately 20 g/d for 5–7 days followed by a maintenance phase of 5 g/d for several weeks [15, 16].

In healthy strength trained humans [17–19], creatine monohydrate supplementation has been shown to improve performance [14, 20], force output [20–22], and muscle free mass [13, 14, 23, 24]. In particular, creatine is an ergogenic aid (i.e., a temporal energy buffer) when supplementation is associated with high intensity exercise and the effect is more pronounced in untrained versus trained and in elderly versus young individuals [25], whereas similar changes in muscle performance have been found in males and females [14, 20]. The accepted mechanism explaining the positive effect of creatine supplementation on performance consists of the temporal energy buffering due to the enhancement of the resting high energy phosphate levels (total creatine, phosphocreatine, creatine, and ATP), leading to a better match between ATP supply and the muscle fibers demands during physical exercise [26]. This change allows users to improve performance through increased total training volume. Creatine monohydrate supplementation is also well known for being responsible of a hypertrophic response determining an increased fat-free mass of about one kilogram [13, 14, 23, 24]. The hypertrophic potential following creatine administration has been mostly linked to fluid retention in myofibers due to swelling-induced osmotic potential of high intracellular creatine [24, 27]. An increased expression of myosin heavy chain isoforms [28] and myogenic regulatory factors [29, 30] and an improved mitotic activity of satellite cells have also been considered as key determinants of the net protein deposition following supplementation. The overall effects of creatine monohydrate supplementation on muscle structure have prompted researchers to investigate its efficacy in treating exercise-induced muscle injuries [26]. So far, contradictory results have been reported. While some studies on animal models and humans show that creatine supplementation does not decrease muscle damage or enhance recovery after high intensity eccentric contractions [31, 32], others have shown contradicting results as a greater isokinetic and isometric strength and a quicker amelioration of plasma creatine kinase (CK) levels during recovery were observed following creatine supplementation from an exercise-induced muscle damage [33]. Given the conflicting results from experiments investigating the efficacy of creatine treatment on skeletal muscle damage and recovery from eccentric-exercise damage we, and others [26], encourage further research to understand what, if any, role creatine treatment can play. Importantly, an anti-inflammatory effect of creatine has been observed when its supplementation was used in runners (with previous experience in running marathons) before a long distance race [34]. This protective effect has been confirmed in double blind trials when creatine supplementation (20 g d-1) was administered before high intensity endurance competitions [35, 36]. It is possible that the benefits of creatine supplementation in preventing muscle damage may relate to its antioxidant potential in endurance settings [26]. However, few studies have been published on the relationship between supplementation and oxidative stress, and controversial and inconclusive results have been obtained on indicators of oxidative damage [37–39]. Accordingly, creatine supplementation has been associated with either no change of lipid peroxidation, resistance of low density lipoprotein to oxidative stress or plasma concentrations of nonenzymatic antioxidants [39], increased free radical generation [37], and reduced oxidative stress [38]. In particular, Rahimi in [38] found a significant increase in athletic performance combined with attenuation of plasma malondialdehyde and urinary 8-hydroxy-2-deoxyguanosine levels in men who underwent 7 days creatine monohydrate supplementation (20 g/d) before a resistance exercise protocol. These results suggested a reduced training-induced oxidative stress and lipid peroxidation associated with supplementation [38]. However, no change [39] or an increase [37] in lipid peroxidation, resistance of low density lipoprotein to oxidative stress and plasma concentrations of nonenzymatic antioxidants has also been reported in adult males performing exhaustive incremental exercise trials combined with creatine supplementation. Taken together, these observations show that creatine supplementation may help in maintaining muscle integrity after intense and prolonged exercise, yet the mechanisms underlying the protective effect are only partially known.

The effects of creatine supplementation on muscle performance and protein metabolism and, possibly, muscle integrity may represent a rationale for its potential use to prevent or treat muscle disorders. Creatine monohydrate supplementation may benefit muscle disorders in a nonspecific fashion [40] by enhancing muscle strength and mass, reducing intracellular calcium accumulation and apoptosis [41], preventing oxidative stress, and attenuating cell death [42]. In fact, in myopathy, pathophysiological events lead to fiber necrosis, apoptosis, autophagy elevation in reactive oxygen species, mitochondrial dysfunction, increases in protein catabolism and degradation, and rise in intracellular calcium content [43], and all these elements may represent sites of attack for creatine. Furthermore a reduced creatine disposal may characterize neuromuscular disorders [44–49], due to the lower creatine transporter content or the impairment of energy charging capacity of the cells. This condition of relative creatine deficiency may boost the need for dietary supplementation. The efficacy of creatine supplementation in muscle diseases has been observed in animal models and humans. For instance, in mdx mice (a model of dystrophinopathy), an improved calcium handling resulting in lower intracellular calcium concentrations and enhanced cell survival have been shown in cultured cells [50]. In the same animal model an improvement of mitochondrial respiration and muscle function has been also observed [51]. In 1997, a prospective randomized study reporting the positive effects of creatine monohydrate supplementation in a neuromuscular disease (i.e. mitochondrial myopathy) was first published [52]. In this study creatine monohydrate was administered in 7 mitochondrial cytopathy patients using a randomized crossover design with the following scheme: 5 g for the first 14 days followed by 2 g of oral creatine monohydrate for the subsequent 7 days. Measurements included activities of daily living, isometric handgrip strength, basal and postexercise lactate, evoked and voluntary contraction strength of the dorsiflexors, and aerobic capacity at cycle ergometry. Creatine treatment resulted in significantly increased handgrip strength, with no changes in the other measured variables [52]. In 1999, Tarnopolsky and Martin [53] found an increase in high intensity power output following creatine monohydrate supplementation for 10 days (10 g daily for 5 days followed by 5 g daily for 5 days) in a heterogeneous group of people with neuromuscular disorders enrolled in two studies (Study 1, *n* = 81 open study; and Study 2, *n* = 21 single-blinded study). In the open trial, which aimed to test the clinical efficacy of creatine supplementation in a large cohort of patients, myopathies included muscle dystrophies (*n* = 15), mitochondrial cytopathies (*n* = 17), inflammatory myopathies (*n* = 14), and peripheral neuropathy disorders (*n* = 18), whereas the single-blinded study was conducted only on 21 heterogeneous patients (*n* = 16, miscellaneous myopathies, *n* = 6 muscle dystrophies, *n* = 1 peripheral neuropathy disorder, and *n* = 1 inflammatory myopathy [53].

Despite the results from [53] being based on a limited number of patients, the acquired knowledge has encouraged further research aimed at testing the effects of creatine supplementation in several conditions including sarcopenia of ageing [54, 55], dystrophies [56, 57], mitochondrial myopathies [58, 59], COPD [60–62], and chronic heart failure (where phosphocreatine/ATP ratio is a stronger prognostic factor than the degree of impairment [63]). To date, the results across all these studies remain inconclusive about a beneficial effect of creatine supplementation.

Meta-analyses of the growing literature base [40, 64, 65], highlighting the statistical under power of the majority of the studies, reveal that the efficacy for long- and short-term supplementation with creatine monohydrate is only seen in selected muscular dystrophies (i.e., dystrophinopathies and myotonic dystrophy type 2 and inflammatory myopathies, such as dermatomyositis and polymyositis) and in terms of increased muscle strength even under anti-inflammatory therapies (corticosteroids). Critical analysis of the literature shows we cannot draw safe conclusions in other myopathic conditions, partly due to methodological biases in setting up clinical trials and small sample sizes leading to lower power [43]. Importantly, in all analyzed studies across all tested myopathic conditions, creatine appeared well tolerated, apart from treatment of glycogenosis type V (McArdle disease). In this case treatment by creatine supplementation at high dose rates resulted in impaired activities of daily living and increased muscle pain (cramping) [43].

Our review of creatine supplementation has highlighted three important points. First, creatine monohydrate supplementation in healthy and diseased humans can have important beneficial effects. Second, conclusions from the study of creatine supplementation in several myopathic conditions are deeply hampered by methodological problems including difficulties in statistical power due to rarity of diseases. Third, there are virtually no observed negative effects of creatine supplementation compared to currently available chemotherapeutic interventions. Together, these three points strongly justify ongoing efforts in establishing basic research, randomized clinical trials, or other experimental designs on testing the benefits of creatine supplementation.

## 3. L-Carnitine

### 3.1. The Nutritional Biochemistry of L-Carnitine

**Figure 2:**
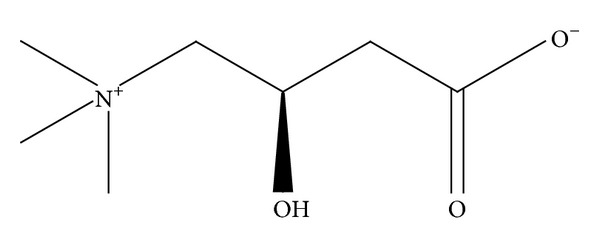
Chemical structure of L-carnitine.

L-Carnitine (3-hydroxy-4-N-trimethylaminobutyrate, Figure 2), the bioactive form of carnitine, is an endogenous branched nonessential amino acid derivative. L-carnitine is synthesized in kidney, liver, and testes, starting from L-lysine and L-methionine, having ascorbic acid, ferrous iron, pyroxidine, and niacin as cofactors. L-carnitine can also be consumed with diet, especially with foods of animal origin. Omnivores have a dietary intake of carnitine from 20 and 300 mg/d mostly from red meat (50–150 mg/100 g), fish, and dairy products (up to 10 mg/100 g) consumption, whereas vegetarians have a dietary intake of about 1–3 mg/d. Dietary carnitine is absorbed in the small intestine and enters the bloodstream [66]. Inside the cells, carnitine is involved in lipid metabolism since it allows the transport of fatty acids with more than 14 carbon atoms from the cytoplasm to mitochondria where they undergo *β*-oxidation [63, 67]. The transition takes place through three steps. The first step is catalysed by carnitine palmitoyl transferase 1 (CPT1) and the transmembrane transport is facilitated by acylcarnitine transferase. Within the mitochondrion, free carnitine is regenerated by the action of carnitine palmitoyl transferase 2 (CPT2) and the released fatty acyl-CoAs enter the *β*-oxidation pathway. Taking into account that free CoA is involved in the pyruvate dehydrogenase reaction and in the process of *β*-oxidation, carnitine contributes to the coordinated integration of fat and carbohydrate metabolism. When glucose oxidation increases, acetyl groups can be translocated from acyl-CoA within the mitochondrial matrix to the cytoplasm. The accumulation of cytosolic acetylcarnitine may result in a limitation of CPT-1 activity because of the decrease in availability of free carnitine [26]. Moreover, fatty acid oxidation could occur, considering that skeletal muscle predominantly expresses an isoform of CPT-1 with low affinity for L-carnitine [68]. Thus, the regulation of free fatty acids *β*-oxidation occurs through the regulation of their mitochondrial content due to leakage of acyl and acetyl moieties leading to a modification of the ratio between esterified carnitine and free carnitine.

Considering its fundamental role in lipid metabolism, L-carnitine is a drug approved by the Food and Drug Administration to treat primary and selected secondary carnitine-deficiency syndromes [69] and is widely used as food supplement for its potential positive effect on health [70] even if the results across available studies remain inconclusive about a real beneficial effect to treat chronic complaints as type 2 diabetes [71] and Alzheimer's neurodegenerative disease [72].

Importantly, lines of evidence suggest positive effects of carnitine supplementation in cardiovascular diseases. A recent meta-analysis revealed that carnitine administration leads to 27% reduction in all-cause mortality, 65% reduction in ventricular arrhythmias, and 40% reduction in anginal symptoms in patients experiencing an acute myocardial infarction. Therefore L-carnitine and propionyl-L-carnitine can be used along with conventional treatment in presence of stable angina, thus contributing to secondary prevention of cardiovascular diseases [73]. Interestingly promising results have been also obtained in presence of intermittent claudication, the most frequent symptom of mild moderate peripheral vascular disease, as propionyl-L-carnitine supplementation can help reducing symptoms and is associated with significant amelioration of functional impairment [74].

### 3.2. The Effects of L-Carnitine Supplementation on Muscle Diseases

The skeletal muscle is the most relevant depository of carnitine, and its availability is critical for the physiological bioenergetics of this tissue. Carnitine deficiency greatly affects skeletal muscle function as found in presence of primary and secondary deficiencies. Primary carnitine deficiency (OMIM 212140), which affects between 1 : 37.000–1 : 100.000 newborn individuals, is an autosomal recessive disorder of fatty acid oxidation resulting from defective carnitine transport caused by mutations in carnitine transporter gene SLC22A5 [75] coding for OCTN2 transport protein. The disease, characterized by very low level of free and total carnitine (free carnitine 1–5 *μ*M and normal 20–55 *μ*M), may have a predominant metabolic or cardiac presentation. The metabolic presentation usually before 2 years of age is characterized by frequent gastrointestinal and respiratory infections, lethargia, hepatomegaly, hypoketotic hypoglycemia, hyperammonemia, and serum creatine kinase elevation [76]. Later cardiomyopathy and hypotonia dominate the medical case [77]. Secondary carnitine deficiency is commonly associated with hemodialysis. In chronic renal insufficiency, undialyzed patients total carnitine, free carnitine, and acylcarnitine accumulate in body tissues due to reduced renal clearance [78, 79]. Besides, following regular hemodialysis, a significant creatine loss arises as demonstrated by reduction of creatine content in serum and skeletal muscle during the dialysis session [80] which is not compensated by endogenous synthesis [79]. Considering that the dialysate carnitine content before and after hemodialysis is far below that of control subjects and that the loss of carnitine into the dialysate greatly exceeds that into urine, the net loss of carnitine is mostly attributed to dialysis procedures [81, 82]. Indeed also the carnitine cofactors and precursors, vitamin B6, niacin, vitamin C, lysine, and methionine, may be lost throughout the dialysis procedures [83]. Considering the role of carnitine in the cell, bioenergetics, dyslipidemia, muscle fatigue, cardiomyopathy, and anemia have been considered potential targets for L-carnitine supplementation in several trials conducted in a large cohort of hemodialyzed patients [83]. Meta-analysis of the literature does not allow to draw consensus on whether carnitine supplementation can improve the patient's health status. In particular, although initial systematic reviews of published literature on the topic put forward a promising effect on management of anemia and failed to demonstrate a significant efficacy in controlling dyslipidemia [83], a recent meta-analysis involving more than 1700 participants failed to confirm the previous findings regarding the effects of L-carnitine on hemoglobin but showed that L-carnitine significantly decreases serum low-density lipoprotein (LDL) and C-reactive protein (CRP). In this study the extent of LDL decrease did not appear clinically relevant, whereas a significant and clinically relevant decrease of CRP serum content was observed [84]. Importantly, these effects were not confirmed in another meta-analysis of randomized controlled trials, which failed to demonstrate any improvement of inflammation, oxidative stress, nutrition, anemia, dyslipidemia, hyperparathyroidism status, or quality of life in hemodialyzed patients [85]. Uncertainties also regard the effect of L-carnitine administration on skeletal muscle function of hemodialyzed patients. Long-term administration (12 months) of L-carnitine (2 g/day) to hemodialyzed patients resulted in increased serum and muscle carnitine levels, and selective type 1 fiber hypertrophy [86] and similar results have been obtained in uremic patients following 24 weeks administration of the same dose [87] but the functional significance of such changes remains to be elucidated. Taken together, available inconclusive results put forward that high-quality and long-term randomized trials are still required to fully elucidate the clinical value of L-carnitine administration in these patients.

Other clinically relevant conditions may determine secondary carnitine deficiency, that is, intestinal resection, severe infections, liver disease, and cancer [88] where a negative impact on skeletal muscle is demonstrated by the appearance of pathological manifestations, including fibers accumulation of neutral lipids, structural damages, and subsarcolemmal accumulation of large aggregates of mitochondria. Therefore, carnitine supplementation may represent a useful tool for the management of muscle deterioration and the appearance of fatigue in presence of carnitine loss.

Carnitine deficiency is not the only condition that allows to focus on its central role in muscle energy disposal and handling. Carnitine availability may be the limiting factor for fatty acid oxidation and/or the removal of acyl-CoAs at rest and during low intensity exercise [89], and an increase in skeletal muscle total carnitine content would be expected to increase fatty acid oxidation and decrease pyruvate dehydrogenase complex activation and glycogen use during such exercise tasks. On the other side, during high intensity exercise, carnitine shifts towards acetylcarnitine formation, thus maintaining the pyruvate dehydrogenase complex and and tricarboxylic acid flux [90]. In accordance with a dual role of carnitine in skeletal muscle energetics, beneficial effect of L-carnitine supplementation has been observed at low intensity exercise where its availability increases the rate of oxidation of intramuscular fatty acids and triacylglycerols, thus postponing the appearance of fatigue [91, 92]. Furthermore at high intensity exercise its availability may lead to better matching of glycolytic and mitochondrial flux, thus reducing ATP formation by anaerobic mechanisms [90, 93–97]. Importantly, some studies failed to observe such effects [98–104], and inconsistencies may be laid on the relevance of dietary means to carnitine retention after supplementation, being favored by carbohydrates coingestion through increased insulin level [90].

One of the most promising areas of research on L-carnitine supplementation regards its potential role in ameliorating and accelerating recovery from exercise-induced muscle injury. It has been found that supplemental carnitine is effective in attenuating signs of tissue damage (muscle soreness and serum CK elevation) induced by lengthening or intense contractions [105–108] also in sarcopenic muscle [109]. The observed benefits of L-carnitine supplementation in preventing load-induced muscle injury have been attributed to its known role as antioxidant. In skeletal muscle, reactive oxygen species (ROS) and nitrogen species are physiologically synthesized at low levels and are required for normal force production [110]. When ROS production overtakes tissue antioxidant capacity, oxidative stress activates pathophysiologic signaling leading to proteolysis and apoptosis within the myofibers. This sequence of events is considered as a major cause of sarcolemmal damage and leakage of cytosolic proteins as CK into the circulation and the origin of reduced muscle strength capacity that contributes to fatigue [111–113]. In exercise-induced muscle damage L-carnitine supplementation has been found to reduce postexercise serum CK [105, 106] and myoglobin concentrations [106], suggesting a quicker muscle recovery from damage. Further evidence demonstrated that L-carnitine has an effective free-radicals scavenging activity at least* in vitro* [114]. In contrast inconclusive results have been obtained* in vivo* at least in humans on the effects of L-carnitine on xanthine oxidase, a marker of metabolic stress that, in presence of high glycolytic rates, mediates the oxidation of AMP to hypoxanthine [115]. Accumulation of xanthine oxidase is the consequence of the activation of calcium-dependent proteases, which cleave a portion of xanthine dehydrogenase and convert it into xanthine oxidase. This response appears to be attenuated by L-carnitine supplementation which reduces intracellular hypoxanthine and xanthine oxidase following resistance exercise bouts [109] whereas other experimental investigations failed to demonstrate such effect [116, 117].

Another mechanism, by which high intensity muscle contractions may exert toxic effect on skeletal muscle, is transient hypoxia. Under hypoxia, an increased concentration of blood ammonia and a lower concentration of free carnitine have been found [118, 119]. In this condition supplementation with L-carnitine may prevent ammonia formation through its antioxidant activity. Besides exercise, L-carnitine is known to protect against muscle mitochondrial dysfunctions associated with oxidative stress caused by a series of conditions such as aging, ischemia reperfusion, inflammation, degenerative diseases, carcinogenesis, and drug toxicity,* in vivo* or* in vitro *[120–134]. For instance, studies suggest that cancer cachexia, which includes anorexia, weight loss, muscle loss, skeletal muscle atrophy, anemia, and alterations in carbohydrate, lipid, and protein metabolism [135], is associated with a decrease in intracellular glutathione concentration in the muscle [136–138], and L-carnitine supplementation increases the tumor-induced decrease in muscular glutamate and glutathione levels at least in animal models [136]. Gramignano et al. [139], studying the efficacy of L-carnitine supplementation (6 g/day for 4 weeks) in a population of advanced cancer patients, found a decreased ROS and increased glutathione peroxidase levels. Promising antioxidant activities have been also found following supplementation in patients with nonalcoholic steatohepatitis [140], renal disease [141], and phenylketonuria [142], as well as in several experimental models of oxidative stress [143–146]. Interestingly, recent evidence suggests that carnitine supplementation may also directly act as radical scavenger, thus contributing to protection against statin-induced oxidative muscle damage [146, 147].

In summary, considering its importance in muscle bioenergetics and its antioxidant potential, L-carnitine supplementation may be considered an aid in presence of carnitine deficiency and in skeletal muscle diseases in which oxidative stress and altered fatty acid oxidation mostly contribute to pathophysiology. Despite this potential, further research is needed to conclusively elucidate the mechanisms underlying its protective effects and to establish whether they may also arise in presence of muscle diseases of different origin.

## 4.  *ω*3 Long Chain Polyunsaturated Fatty Acids (*ω*3 LC-PUFAs)

### 4.1. The Nutritional Biochemistry of *ω*3 LC-PUFAs

**Figure 3:**
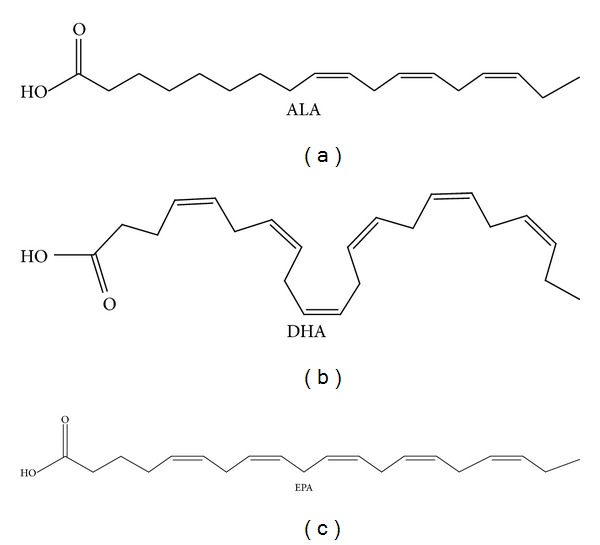
Chemical structure of α-linolenic acid (ALA), docosahexaenoic acid (DHA), and eicosapentaenoic acid (EPA).

*ω*3 PUFAsare among the most studied nutrients which show healthy properties [148]. α-Linolenic acid (ALA—C18:3, *ω*3, Figure 3), which is not synthesized in the human body and therefore must be consumed with the diet, is the precursor of the two most important bioactive long chain polyunsaturated fatty acids (*ω*3 LC-PUFA, Figure 3): eicosapentaenoic acid (EPA—C20:5 *ω*3) and docosahexaenoic acid (DHA—C22:6 *ω*3). In the endoplasmic reticulum, ALA is converted in EPA and DHA through the development of enzymatic elongation and desaturation reactions, in which Δ-6 desaturase and elongase and Δ-5 desaturase enzymes are involved. In peroxisomes, these reactions are followed by *β*-oxidation to produce DHA. *ω*3 LC-PUFAs are considered “conditionally essential” because occasionally they are not synthesized in sufficient amounts to meet human needs. This condition occurs when the dietary intake of ALA is too low in comparison with linoleic acid (LA, C18:2-*ω*6) and the ratio *ω*6/*ω*3 is higher than 5/1. Thus, due to the competition of ALA and LA for the same Δ-6 desaturase enzyme, which is shared in the two metabolic pathways, the production of LC-PUFAs shifts towards the synthesis of *ω*6 fatty acids. It is therefore evident that the intake of both ALA and EPA and DHA should be encouraged. The most important sources of *ω*3 LC-PUFAs in human nutrition are fatty fish, such as sardines, salmon, and tuna, as well as walnuts, and flaxseed, and canola oils.

Once produced from ALA, EPA and DHA, in turn, are precursors of eicosanoids that mediate the anti-inflammatory effects that underlie the beneficial effects ascribed to *ω*3 LC-PUFAs in numerous physiological and pathological states. In particular, increasing evidence suggests that *ω*3 PUFAs improve blood lipid levels (inducing a decrease of triglyceride levels and an increase of HDL cholesterol), reduce arrhythmias and risk of stroke, and help to prevent and treat atherosclerosis [149, 150]. Moreover, *ω*3 PUFAs were shown to have chemopreventive properties against various types of cancer, including colon and breast cancer [151, 152]. Some recent studies suggest that *ω*3 PUFAs intake is associated with reduced depressive symptoms, particularly in females, potentiating the effects of antidepressants, and helps to reduce mood swings [153, 154]. The protective effect of DHA in amyloid-*β* peptide-infused rats was associated with increased membrane fluidity which also provided oxidative stress resistance in hippocampal cells [155, 156].* In vivo*, *ω*3 LC-PUFAs increased membrane fluidity in rat hippocampus and improved memory formation, whereas their reduction exerted opposite effects [157, 158]. Preclinical studies supported the idea that DHA maintained membrane fluidity, improved synaptic and neurotransmitter functioning, enhanced learning and memory performances, and displayed neuroprotective properties [159]. Moreover, DHA decreased the amount of vascular amyloid-*β* peptide (A*β*) deposition and reduced A*β* burden [160, 161].

Interesting avenues of research also regard the treatment of chronic inflammatory diseases of the bone as rheumatoid arthritis [162, 163]. Importantly, *ω*3 PUFAs have shown beneficial effects on bone health in animal studies [164, 165] but current research suggests only a modest increase in bone turnover in humans [166].

### 4.2. The Effects of *ω*3 PUFA Supplementation on Muscle Diseases

Research on the role of PUFAs in muscle health and functionality is still incomplete and deserves future in-depth analysis. Promising observations suggest an important role of dietary *ω*3 PUFA supplementation on protein synthesis and inflammation and its potential efficacy in lean body mass sparing. Initial evidence suggests that fish-oil-derived *ω*3 PUFAs might be useful in preventing and treating sarcopenia of ageing. This effect appears mediated by increased insulin levels and amino acid mediated activation (i.e. phosphorylation) of signaling proteins of the mTOR/p70S6 K1 pathway, as demonstrated in animal models (i.e. growing steers and burned guinea pigs) [167, 168]. In 2011, Smith et al. in two randomized studies [169–171] demonstrated the effect of *ω*3 PUFA supplementation (1.86 g EPA and 1.50 g DHA for 8 weeks) on muscle protein metabolism in young/middle-aged (mean age: 37 years) and elderly subjects (mean age: 65 years) of both sexes. Importantly, in all individuals, *ω*3 PUFA supplementation, although not exerting any effect on the basal rate of protein synthesis, determined the augmentation of the hyperaminoacidemia-hyperinsulinemia-induced rate. This change was accompanied by increased phosphorylation of mTOR in serine 2448 and downstream p70S6 K in threonine 389, whereas no change in the level of Akt (the main effector of insulin activation upstream mTOR) phosphorylation was observed. Interestingly, the effect on protein synthesis was not associated with lower plasma concentration of inflammatory markers and triglycerides, thus suggesting that the anabolic effect of PUFAs may arise independently of their known anti-inflammatory effect [169]. Furthermore the observed anabolic effect did not produce significant amelioration of glycemic control through changes in skeletal muscle insulin sensitivity, which is generally, but not universally [172–175], accepted to be improved by *ω*3 PUFA supplementation. In fact, Liu et al.[172] showed that in rats fed with a high-*ω*3 fatty acid diet there is an increase of insulin binding to sarcolemma due to changes of the fatty acyl composition of phospholipids surrounding the insulin receptor. The authors suggested that this might be the mechanism by which dietary fatty acids modify insulin action. More recently, in rats fed with a high-saturated fat diet, Holness et al. [173] reported that hyperinsulinemia can be rapidly reversed via the dietary provision of small amounts of *ω*3 PUFA. However, the saving of insulin induced by *ω*3 PUFA supplementation occurs in the absence of an acute improvement of insulin sensitivity. These results are not surprising as the published available information on the effects of PUFA supplementation on insulin sensitivity in humans is inconsistent and often contradictory [176–180].

Considering that maintenance of muscle mass is a fundamental determinant of its capacity to generate force, of interest is the potential correlation between PUFAs consumption and muscle strength in the elderly. So far, inconclusive results are available [180, 181]. In particular, in 2009, a cross-sectional study found no correlation between total *ω*3 or *ω*6 PUFA intakes and muscle strength in aged Americans [180], whereas, in 2013, the Tokyo Oldest Old Survey on Total Health showed that higher consumption of EPA and DHA is significantly associated with higher functional mobility in men. This effect was not observed in women [181]. The only available randomized double blind pilot study analyzed the effects of 2 fish oil (1.2 g EPA and DHA) or 2 placebo (olive oil) capsules per day for 6 months in 126 postmenopausal women [182]. Fatty acid levels, frailty assessment, hand grip strength, 8-foot walk, body composition, and inflammatory biomarkers were taken at baseline and after 6 months of supplementation. Fish oil supplementation resulted in higher red blood cell DHA, compared to baseline and placebo, and improvement in walking speed compared to placebo. In this work a linear regression model including age, vitamin intake, osteoarthritis, frailty phenotype, and tumor necrosis factor alpha (TNF-α) explained that the change in DHA/arachidonic ratio, TNF-α, and selenium intake had the major contribution to the observed change in walking speed [182]. Importantly, new avenues of research highlight that a fundamental booster of the functional effects of fish oil supplementation is physical exercise and, in particular, strength training. In 2012, Rodacki and coworkers [183], in a randomized study enrolling elderly women, demonstrated that the use of fish oil for 90 days in addition to strength training (3 times/week, for 12 weeks, 36 training sessions) was associated with an additional increase of peak torque and rate of torque development, which,* de facto*, defines an improvement in whole body functional capacity as demonstrated by higher chair-rising performance. Notably, the mechanisms underlying these changes remain obscure and the potential role of fish oil in changing the fluidity of the muscle fibers membrane and acetylcholine sensitivity should be taken into consideration [183].

Therefore, available data suggest a potential role of *ω*3 PUFA supplementation in muscle anabolism and functionality at least in presence of sarcopenia of ageing. Future studies are needed to investigate whether these effects might be quantitatively relevant in other myopathic conditions.

Another fundamental mechanism to be taken into consideration when analyzing the potential benefit of *ω*3 PUFA supplementation in healthy and diseased skeletal muscle is the largely demonstrated inhibitory effect on inflammation [184]. *ω*3 PUFAs serve as precursors to prostaglandins such as prostaglandin E3, which are powerful hormone-like substances that reduce inflammation [185], and inhibit arachidonic acid, derived 2-series prostaglandins, and the 4-series leucotrienes, which are known to modulate the production of proinflammatory and immunoregulatory cytokines [186].* In vivo* and* ex vivo* animal studies have indicated that *ω*3 PUFAs reduce the production of TNF-α, IL-1, IL-2, and IL-6 [187–189]. In humans contradictory observations still exist, since some lines of evidence suggest that supplementation of the diet with fish oils results in a reduced production of IL-1, IL-6, TNF-α, and IL-2 [190], and an alteration of gene expression profiles to a more antiinflammatory and antiatherogenic status in peripheral blood mononuclear cells* in vitro* [191]; whereas,* in vivo*, dietary supplementation with PUFAs produced either no change [192] or amelioration of hallmarks of muscle damage (i.e., soreness and creatine kinase levels) and inflammatory mediators (i.e. IL6), following physiological proinflammatory events such as strenuous exercise or eccentric contractions in unaccustomed individuals [193, 194]. Finally, the change in lipid composition of the membranes (i.e. the sarcolemmal membranes) might be considered an additional, not enough explored, effect of PUFA supplementation on skeletal muscle. It is well known that changes in lipid composition may influence membrane function by regulating protein and lipid membrane homeostasis [195–197], and PUFAs either as constituents of membrane phospholipids or free molecules contribute to membrane chemophysical features (i.e., membrane organization, ion permeability, elasticity, and microdomain formation). In particular, it has been shown that *ω*3 PUFA supplementation decreases membrane thickness [198, 199], modulates proton membrane permeability and leaflet thickness enhancing fatty acid flip-flop rate, and increases bilayer propensity to be in a liquid-disordered phase [196]. The anti-inflammatory potential of *ω*3 PUFAs together with their efficacy in modulating myocyte membrane composition and conformation [197, 200–202] was the rational basis for recent preliminary attempts aiming at identifying new strategies for the treatment of cancer cachexia and muscular dystrophies where inflammation plays a major role in the pathogenesis of muscle wasting [203].

Unfortunately, only few and contradictory studies are available. Inconclusive results are available on whether the anti-inflammatory effects of PUFAs can counteract the action of proinflammatory cytokines involved in the pathogenesis of cancer cachexia [203], and the available randomized controlled clinical trials could not safely demonstrate positive effects of supplementation on muscle wasting [204]. Few and contradictory results have also been obtained in muscular dystrophies. In particular, Fiaccavento et al. have demonstrated that supplementation of dystrophic UM-X7.1 hamsters, carrying a phenotype similar to limb girdle muscular dystrophy 2F (LGMD2F) with ALA, precludes myocyte and muscular tissue damage and modulates cells proliferation promoting myogenic differentiation. Two major factors concurred to determine such effects including the modulation of the lipid membrane composition and configuration which appears altered in such model [205] associated with the preservation of the expression and location of key-role signaling proteins (i.e. *β*-catenin, caveolin-3, sarcoglycan, and dystroglycan) and the slowing down of the myocyte degeneration/regeneration cycling rate associated with the enhancement of the myogenic differentiation [206]. Further, initial observations suggest that *ω*3 PUFA supplementation with fish oil or EPA decreases muscle degeneration and inflammation in mdx mouse model mirrored by reduced functional impairment evaluated by grip strength tests [207, 208]. On the contrary, by using a highly controlled diet design, Henderson et al. [209] have recently put forward a detrimental effect of a high intake of *ω*3 PUFA, unlike high MUFA, in the same animal model as demonstrated by higher serum CK activity and no changes in skeletal muscle histopathology and inflammatory markers (p65) [209]. Congruent with the latter observations, Galvao and coworkers [210] have recently demonstrated that high *ω*3-PUPA diet enriched with α-linoleic and α-linolenic acid, unlike high long chain saturated fatty acids diet, promotes a negative effect on lifespan in the same animal model with genetic cardiomyopathy. Importantly the harmful effect on survival of the high PUFA diet appeared to be associated with highly significant increase in plasma free fatty acids whose elevation is correlated with higher risk of ventricular arrhythmias and is considered a strong predictor of sudden cardiac death [211, 212].

Therefore, considering the available research studies, it is not currently possible to draw safe conclusions on the role of *ω*3 PUFA in muscle diseases and further studies are first needed to elucidate whether supplementation may be harmful in certain myopathic conditions.

## 5. Conclusion

Myopathies are chronic degenerative diseases that induce the deterioration of the structure and function of skeletal muscle characterized by progressive strength loss, muscle fatigue, pain, or tenderness, cramps, stiffness, and tightness. So far, curative therapies are not available and the goals of treatment are now finalized to delay the onset of the disease and to relieve symptoms. Therefore, myopathic patients are submitted to interventions, such as rehabilitation, ventilatory assistance, and nutritional approach, which have the main purposes of improving the quality of life and slowing the progression of the disease. In particular an adequate diet, which has sufficient caloric content and balanced nutritional composition, and specific nutritional supplements can help myopathic patients. The presented review of the literature suggests that creatine, L-carnitine, and *ω*3-PUFA supplementation may have the potential to exert beneficial effects in selected myopathies. Nevertheless, to date, experimental evidence of short-term and long-term effects of supplementation in myopathies with heterogeneous physiopathology is missing. Indeed, the evidence of potential harmful effects in short-term settings (*ω*3 PUFA in animal models of dystrophy and cardiomyopathy and creatine in McArdle disease) and the lack of studies on the potential harmful effects of prolonged supplementations strongly highlight that further rigorous studies are required before these supplementations could be recommended as a treatment in selected muscle diseases.

## References

[1] Nabavi SM, Daglia M, Moghaddam AH, Nabavi SF, Curti V (2014). Tea consumption and ischemic stroke: a brief review of the literature. *Current Pharmaceutical Biotechnology*.

[2] Daglia M, Di Lorenzo A, Nabavi SF, Talas ZS, Nabavi SM Gallic acid and related compounds as neuroprotective agents: you are what you eat!.

[3] Garcìa-Lafuente A, Moro C, Manchòn N (2014). In vitro anti-inflammatory activity of phenolic rich extracts from white and red beans. *Food Chemistry*.

[4] Flachs P, Rossmeisl M, Kopecky J (2014). The effect of n-3 fatty acids on glucose homeostasis and insulin sensitivity. *Physiological Research*.

[5] Aartsma-Rus A, van Ommen GJ, Kaplan J (2013). Innovating therapies for muscle diseases. *Handbook of Clinical Neurology*.

[6] Wyss M, Kaddurah-Daouk R (2000). Creatine and creatinine metabolism. *Physiological Reviews*.

[7] Branch JD (2003). Effect of creatine supplementation on body composition and performance: a meta-analysis. *International Journal of Sport Nutrition and Exercise Metabolism*.

[8] Jäger R, Purpura M, Shao A, Inoue T, Kreider RB (2011). Analysis of the efficacy, safety, and regulatory status of novel forms of creatine. *Amino Acids*.

[9] Kato T (1994). Reduction of brain phosphocreatine in bipolar II disorder detected by phosphorus-31 magnetic resonance spectroscopy. *Journal of Affective Disorders*.

[10] Chaturvedi RK, Flint Beal M (2013). Mitochondrial diseases of the brain. *Free Radical Biology and Medicine*.

[11] Ravina B (2006). A randomized, double-blind, futility clinical trial of creatine and minocycline in early Parkinson disease. *Neurology*.

[12] Kieburtz K, Tilley B, Ravina B (2008). A pilot clinical trial of creatine and minocycline in early Parkinson disease: 18-Month results. *Clinical Neuropharmacology*.

[13] Hultman E, Söderlund K, Timmons JA, Cederblad G, Greenhaff PL (1996). Muscle creatine loading in men. *Journal of Applied Physiology*.

[14] Harris RC, Soderlund K, Hultman E (1992). Elevation of creatine in resting and exercised muscle of normal subjects by creatine supplementation. *Clinical Science*.

[15] Green AL, Hultman E, Macdonald IA, Sewell DA, Greenhaff PL (1996). Carbohydrate ingestion augments skeletal muscle creatine accumulation during creatine supplementation in humans. *The American Journal of Physiology—Endocrinology and Metabolism*.

[16] Schoch RD, Willoughby D, Greenwood M (2006). The regulation and expression of the creatine transporter: a brief review of creatine supplementation in humans and animals. *Journal of the International Society of Sports Nutrition*.

[17] van Loon LJC, Oosterlaar AM, Hartgens F, Hesselink MKC, Snow RJ, Wagenmakers AJM (2003). Effects of creatine loading and prolonged creatine supplementation on body composition, fuel selection, sprint and endurance performance in humans. *Clinical Science*.

[18] Van Schuylenbergh R, Van Leemputte M, Hespel P (2003). Effects of oral creatine-pyruvate supplementation in cycling performance. *International Journal of Sports Medicine*.

[19] Izquierdo M, Ibañez J, González-Badillo JJ, Gorostiaga EM (2002). Effects of creatine supplementation on muscle power, endurance, and sprint performance. *Medicine and Science in Sports and Exercise*.

[20] Tarnopolsky MA, MacLennan DP (2000). Creatine monohydrate supplementation enhances high-intensity exercise performance in males and females. *International Journal of Sport Nutrition*.

[21] Casey A, Constantin-Teodosiu D, Howell S, Hultman E, Greenhaff RL (1996). Creatine ingestion favorably affects performance and muscle metabolism during maximal exercise in humans. *The American Journal of Physiology*.

[22] Terjung RL, Clarkson P, Eichner ER (2000). The American College of Sports Medicine Roundtable on the physiological and health effects of oral creatine supplementation. *Medicine and Science in Sports and Exercise*.

[23] Mihic S, MacDonald JR, McKenzie S, Tarnopolsky MA (2000). Acute creatine loading increases fat-free mass, but does not affect blood pressure, plasma creatinine, or CK activity in men and women. *Medicine and Science in Sports and Exercise*.

[24] Safdar A, Yardley NJ, Snow R, Melov S, Tarnopolsky MA (2008). Global and targeted gene expression and protein content in skeletal muscle of young men following short-term creatine monohydrate supplementation. *Physiological Genomics*.

[25] Tarnopolsky MA (2000). Potential benefits of creatine monohydrate supplementation in the elderly. *Current Opinion in Clinical Nutrition and Metabolic Care*.

[26] G. D’Antona, G. N. Bisciotti (2013). Nutritional interventions as potential strategy to minimize exercise-induced muscle injuries in sports. *Muscle Injuries in Sport Medicine*.

[27] Deldicque L, Atherton P, Patel R (2008). Effects of resistance exercise with and without creatine supplementation on gene expression and cell signaling in human skeletal muscle. *Journal of Applied Physiology*.

[28] Willoughby DS, Rosene J (2001). Effects of oral creatine and resistance training on myosin heavy chain expression. *Medicine and Science in Sports and Exercise*.

[29] Willoughby DS, Rosene JM (2003). Effects of oral creatine and resistance training on myogenic regulatory factor expression. *Medicine and Science in Sports and Exercise*.

[30] Hespel P, Eijnde BO, van Leemputte M (2001). Oral creatine supplementation facilitates the rehabilitation of disuse atrophy and alters the expression of muscle myogenic factors in human. *Journal of Physiology*.

[31] Warren GL, Fennessy JM, Millard-Stafford ML (2000). Strength loss after eccentric contractions is unaffected by creatine supplementation. *Journal of Applied Physiology*.

[32] Rawson ES, Gunn B, Clarkson PM (2001). The effects of creatine supplementation on exercise-induced muscle damage. *Journal of Strength and Conditioning Research/National Strength & Conditioning Association*.

[33] Cooke MB, Rybalka E, Williams AD, Cribb PJ, Hayes A (2009). Creatine supplementation enhances muscle force recovery after eccentrically-induced muscle damage in healthy individuals. *Journal of the International Society of Sports Nutrition*.

[34] Santos RVT, Bassit RA, Caperuto EC, Costa Rosa LFBP (2004). The effect of creatine supplementation upon inflammatory and muscle soreness markers after a 30km race. *Life Sciences*.

[35] Bassit RA, Curi R, Costa Rosa LFBP (2008). Creatine supplementation reduces plasma levels of pro-inflammatory cytokines and PGE2 after a half-ironman competition. *Amino Acids*.

[36] Bassit RA, Pinheiro CHDJ, Vitzel KF, Sproesser AJ, Silveira LR, Curi R (2010). Effect of short-term creatine supplementation on markers of skeletal muscle damage after strenuous contractile activity. *European Journal of Applied Physiology*.

[37] Percário S, Domingues SPDT, Teixeira LFM (2012). Effects of creatine supplementation on oxidative stress profile of athletes. *Journal of the International Society of Sports Nutrition*.

[38] Rahimi R (2011). Creatine supplementation decreases oxidative DNA damage and lipid peroxidation induced by a single bout of resistance exercise. *Journal of Strength and Conditioning Research*.

[39] Kingsley MIC, Cunningham D, Mason L, Kilduff LP, McEneny J (2009). Role of creatine supplementation on exercise-induced cardiovascular function and oxidative stress. *Oxidative Medicine and Cellular Longevity*.

[40] Kley RA, Tarnopolsky MA, Vorgerd M (2013). Creatine for treating muscle disorders (Review). *The Cochrane Database of Systematic Review*.

[41] Sullivan PG, Geiger JD, Mattson MP, Scheff SW (2000). Dietary supplement creatine protects against traumatic brain injury. *Annals of Neurology*.

[42] Gualano B, Artioli GG, Poortmans JR, Lancha Junior AH (2010). Exploring the therapeutic role of creatine supplementation. *Amino Acids*.

[43] Tarnopolsky MA (2007). Clinical use of creatine in neuromuscular and neurometabolic disorders. *Sub-Cellular Biochemistry*.

[44] Matthews PM, Arnold DL (1990). Phosphorus magnetic resonance spectroscopy of brain in mitochondrial cytopathies. *Annals of Neurology*.

[45] Matthews PM, Allaire C, Shoubridge EA, Karpati G, Carpenter S, Arnold DL (1991). In vivo muscle magnetic resonance spectroscopy in the clinical investigation of mitochondrial disease. *Neurology*.

[46] Park JH, Olsen NJ, King L (1995). Use of magnetic resonance imaging and P-31 magnetic resonance spectroscopy to detect and quantify muscle dysfunction in the amyopathic and myopathic variants of dermatomyositis. *Arthritis & Rheumatism*.

[47] Tarnopolsky MA, Parise G (1999). Direct measurement of high-energy phosphate compounds in patients with neuromuscular disease. *Muscle and Nerve*.

[48] Tarnopolsky MA, Parshad A, Walzel B, Schlattner U, Wallimann T (2001). Creatine transporter and mitochondrial creatine kinase protein content in myopathies. *Muscle and Nerve*.

[49] Banerjee B, Sharma U, Balasubramanian K, Kalaivani M, Kalra V, Jagannathan NR (2010). Effect of creatine monohydrate in improving cellular energetics and muscle strength in ambulatory Duchenne muscular dystrophy patients: a randomized, placebo-controlled 31P MRS study. *Magnetic Resonance Imaging*.

[50] Pulido SM, Passaquin AC, Leijendekker WJ, Challet C, Wallimann T, Rüegg UT (1998). Creatine supplementation improves intracellular Ca^2+^ handling and survival in mdx skeletal muscle cells. *The FEBS Letters*.

[51] Passaquin AC, Renard M, Kay L (2002). Creatine supplementation reduces skeletal muscle degeneration and enhances mitochondrial function in mdx mice. *Neuromuscular Disorders*.

[52] Tarnopolsky MA, Roy BD, MacDonald JR (1997). A randomized, controlled trial of creatine monohydrate in patients with mitochondrial cytopathies. *Muscle & Nerve*.

[53] Tarnopolsky M, Martin J (1999). Creatine monohydrate increases strength in patients with neuromuscular disease. *Neurology*.

[54] Chilibeck PD, Chrusch MJ, Chad KE, Davison KS, Burke DG (2005). Creatine monohydrate and resistance training increase bone mineral content and density in older men. *Journal of Nutrition, Health and Aging*.

[55] Stout JR, Sue Graves B, Cramer JT (2007). Effects of creatine supplementation on the onset of neuromuscular fatigue threshold and muscle strength in elderly men and women (64–86 years). *Journal of Nutrition, Health and Aging*.

[56] Tarnopolsky M, Mahoney D, Thompson T, Naylor H, Doherty TJ (2004). Creatine monohydrate supplementation does not increase muscle strength, lean body mass, or muscle phosphocreatine in patients with myotonic dystrophy type 1. *Muscle and Nerve*.

[57] Tarnopolsky MA, Mahoney DJ, Vajsar J (2004). Creatine monohydrate enhances strength and body composition in Duchenne muscular dystrophy. *Neurology*.

[58] Klopstock T, Querner V, Schmidt F (2000). A placebo-controlled crossover trial of creatine in mitochondrial diseases. *Neurology*.

[59] Kornblum C, Schröder R, Müller K (2005). Creatine has no beneficial effect on skeletal muscle energy metabolism in patients with single mitochondrial DNA deletions: a placebo-controlled, double-blind 31P-MRS crossover study. *European Journal of Neurology*.

[60] Deacon SJ, Vincent EE, Greenhaff PL (2008). Randomized controlled trial of dietary creatine as an adjunct therapy to physical training in chronic obstructive pulmonary disease. *The American Journal of Respiratory and Critical Care Medicine*.

[61] Faager G, Söderlund K, Sköld CM, Rundgren S, Tollbäck A, Jakobsson P (2006). Creatine supplementation and physical training in patients with COPD: a double blind, placebo-controlled study. *International Journal of Chronic Obstructive Pulmonary Disease*.

[62] Fuld JP, Kilduff LP, Neder JA (2005). Creatine supplementation during pulmonary rehabilitation in chronic obstructive pulmonary disease. *Thorax*.

[63] Rebouche CJ (1986). Synthesis of carnitine precursors and related compounds. *Methods in Enzymology*.

[64] Kley RA, Vorgerd M, Tarnopolsky MA (2007). Creatine for treating muscle disorders. *Cochrane Database of Systematic Reviews*.

[65] Kley RA, Tarnopolsky MA, Vorgerd M (2011). Creatine for treating muscle disorders. *The Cochrane Database of Systematic Reviews*.

[66] Flanagan JL, Simmons PA, Vehige J, Willcox MD, Garrett Q (2010). Role of carnitine in disease. *Nutrition and Metabolism*.

[67] Rebouche CJ, Paulson DJ (1986). Carnitine metabolism and function in humans. *Annual Review of Nutrition*.

[68] Weis BC, Cowan AT, Brown N, Foster DW, McGarry JD (1994). Use of a selective inhibitor of liver carnitine palmitoyltransferase I (CPT I) allows quantification of its contribution to total CPT I activity in rat heart: evidence that the dominant cardiac CPT I isoform is identical to the skeletal muscle enzyme. *The Journal of Biological Chemistry*.

[69] Pons R, De Vivo DC (1995). Primary and secondary carnitine deficiency syndromes. *Journal of Child Neurology*.

[70] Couturier A, Ringseis R, Mooren FC, Kruger K, Most E, Eder K (2014). Correction: Carnitine supplementation to obese Zucker Rats prevents obesità-induced type I to type II muscle fiber transition and favor san oxidative phenotype of skeletal muscle. *Nutrition & Metabolism*.

[71] Ringseis R, Keller J, Eder K (2012). Role of carnitine in the regulation of glucose homeostasis and insulin sensitivity: evidence from in vivo and in vitro studies with carnitine supplementation and carnitine deficiency. *European Journal of Nutrition*.

[72] Mancuso C, Siciliano R, Barone E, Preziosi P (2012). Natural substances and Alzheimer’s disease: from preclinical studies to evidence based medicine. *Biochimica et Biophysica Acta*.

[73] Di Nicolantonio JJ, Lavie CJ, Fares H, Menezes AR, O'Keefe JH (2013). L-carnitine in the secondary prevention of cardiovascular disease: systematic review and meta-analysis. *Mayo Clinic Proceedings*.

[74] Delaney CL, Spark JI, Thomas J, Wong YT, Chan LT, Miller MD (2013). A systematic review to evaluate the effectiveness of carnitine supplementation in improving walking performance among individuals with intermittent claudication. *Atherosclerosis*.

[75] Wang Y, Korman SH, Ye J (2001). Phenotype and genotype variation in primary carnitine deficiency. *Genetics in Medicine*.

[76] Longo N, Amat Di San Filippo C, Pasquali M (2006). Disorders of carnitine transport and the carnitine cycle. *The American Journal of Medical Genetics—Seminars in Medical Genetics*.

[77] El-Hattab AW, Li FY, Shen J (2010). Maternal systemic primary carnitine deficiency uncovered by newborn screening: clinical, biochemical, and molecular aspects. *Genetics in Medicine*.

[78] Chen SH, Lincoln SD (1977). Increased serum carnitine concentration in renal insufficiency. *Clinical Chemistry*.

[79] Rodriguez-Segade S, Alonso de La Pena C, Paz M (1986). Carnitine concentrations in dialysed and undialysed patients with chronic renal insufficiency. *Annals of Clinical Biochemistry*.

[80] Golper TA, Wolfson M, Ahmad S (1990). Multicenter trial of L-carnitine in maintenance hemodialysis patients. I. Carnitine concentrations and lipid effects. *Kidney International*.

[81] Boehmer T, Rydning A, Solberg HE (1974). Carnitine levels in human serum in health and disease. *Clinica Chimica Acta*.

[82] Bohmer T, Bergrem H, Eiklid K (1978). Carnitine deficiency induced during intermittent haemodialysis for renal failure. *The Lancet*.

[83] Hurot JM, Cucherat M, Haugh M, Fouque D (2002). Effects of L-carnitine supplementation in maintenance hemodialysis patients: a systematic review. *Journal of the American Society of Nephrology*.

[84] Chen Y, Abbate M, Tang L (2014). L-Carnitine supplementation for adults with end-stage kidney disease requiring maintenance hemodialysis: a systematic review and meta-analysis. *The American Journal of Clinical Nutrition*.

[85] Yang SK, Xiao L, Song PA, Xu X, Liu FY, Sun L (2014). Effect of L-carnitine therapy on patients in maintenance hemodialysis: a systematic review and meta-analysis. *Journal of Nephrology*.

[86] Spagnoli LG, Palmieri G, Mauriello A (1990). Morphometric evidence of the trophic effect of L-carnitine on human skeletal muscle. *Nephron*.

[87] Giovenali P, Fenocchio D, Montanari G (1994). Selective trophic effect of L-carnitine in type I and IIa skeletal muscle fibers. *Kidney International*.

[88] Silvério R, Laviano A, Fanelli FR, Seelaender M (2011). L-carnitine and cancer cachexia: clinical and experimental aspects. *Journal of Cachexia, Sarcopenia and Muscle*.

[89] Brass EP (2000). Supplemental carnitine and exercise. * The American Journal of Clinical Nutrition*.

[90] Wall BT, Stephens FB, Constantin-Teodosiu D, Marimuthu K, Macdonald IA, Greenhaff PL (2011). Chronic oral ingestion of l-carnitine and carbohydrate increases muscle carnitine content and alters muscle fuel metabolism during exercise in humans. *The Journal of Physiology*.

[91] Arenas J, Huertas R, Campos Y, Diaz AE, Villalon JM, Vilas E (1994). Effects of L-carnitine on the pyruvate dehydrogenase complex and carnitine palmitoyl transferase activities in muscle of endurance athletes. *FEBS Letters*.

[92] Huertas R, Campos Y, Diaz E (1992). Respiratory chain enzymes in muscle of endurance athletes: effect of L-carnitine. *Biochemical and Biophysical Research Communications*.

[93] Brass EP, Hiatt WR (1998). The role of carnitine and carnitine supplementation during exercise in man and in individuals with special needs. *Journal of the American College of Nutrition*.

[94] Dragan IG, Vasiliu A, Georgescu E, Eremia N (1989). Studies concerning chronic and acute effects of L-carnitina in elite athletes. *Revue Roumaine de Morphologie,d'Embryologie et de Physiologie - Serie Physiologie*.

[95] Drǎgan GI, Wagner W, Ploeşteanu E (1988). Studies concerning the ergogenic value of protein supply and 1-carnitine in elite junior cyclists. *Physiologie*.

[96] Vecchiet L, di Lisa F, Pieralisi G (1990). Influence of L-carnitine administration on maximal physical exercise. *European Journal of Applied Physiology and Occupational Physiology*.

[97] Arenas J, Ricoy JR, Encinas AR (1991). Carnitine in muscle, serum, and urine of nonprofessional athletes: effects of physical exercise, training, and L-carnitine administration. *Muscle and Nerve*.

[98] Barnett C, Costill DL, Vukovich MD (1994). Effect of l-carnitine supplementation on muscle and blood carnitine content and lactate accumulation during high-intensity sprint cycling. *International Journal of Sport Nutrition*.

[99] Vukovich MD, Costill DL, Fink WJ (1994). Carnitine supplementation: effect on muscle carnitine and glycogen content during exercise. *Medicine and Science in Sports and Exercise*.

[100] P. Colombani (1996). Effects of L-carnitine supplementation on physical performance and energy metabolism of endurance-trained athletes: a double-blind crossover field study. *European Journal of Applied Physiology and Occupational Physiology*.

[101] Nüesch R, Rossetto M, Martina B (1999). Plasma and urine carnitine concentrations in well-trained athletes at rest and after exercise. Influence of L-carnitine intake. *Drugs under Experimental and Clinical Research*.

[102] Trappe SW, Costill DL, Goodpaste B, Vukovich MD, Fink WJ (1994). The effects of L-carnitine supplementation on performance during interval swimming. *International Journal of Sports Medicine*.

[103] Oyono-Enguelle S, Freund H, Ott C (1988). Prolonged submaximal exercise and L-carnitine in humans. *European Journal of Applied Physiology and Occupational Physiology*.

[104] Karlic H, Lohninger A (2004). Supplementation of L-carnitine in athletes: does it make sense?. *Nutrition*.

[105] Giamberardino MA, Dragani L, Valente R, di Lisa F, Saggini R, Vecchiet L (1996). Effects of prolonged L-carnitine administration on delayed muscle pain and CK release after eccentric effort. *International Journal of Sports Medicine*.

[106] Volek JS, Kraemer WJ, Rubin MR, Gómez AL, Ratamess NA, Gaynor P (2002). Carnitine L-tartrate supplementation favorably affects markers of recovery from exercise stress. *The American Journal of Physiology—Endocrinology and Metabolism*.

[107] Kraemer WJ, Volek JS, French DN (2003). The effects of L- carnitine L-tartrate supplementation on hormonal responses to resistance exercise and recovery. *Journal of Strength and Conditioning Research / National Strength & Conditioning Association*.

[108] Spiering BA, Kraemer WJ, Vingren JL (2007). Responses of criterion variables to different supplemental doses of L-carnitine L-tartrate. *Journal of Strength and Conditioning Research*.

[109] Ho J, Kraemer WJ, Volek JS (2010). L-Carnitine l-tartrate supplementation favorably affects biochemical markers of recovery from physical exertion in middle-aged men and women. *Metabolism: Clinical and Experimental*.

[110] Reid MB (2001). Nitric oxide, reactive oxygen species, and skeletal muscle contraction. *Medicine and Science in Sports and Exercise*.

[111] Reid MB, Haack KE, Franchek KM, Valberg PA, Kobzik L, West MS (1992). Reactive oxygen in skeletal muscle. I. Intracellular oxidant kinetics and fatigue in vitro. *Journal of Applied Physiology*.

[112] Westerblad H, Allen DG (2003). Cellular mechanisms of skeletal muscle fatigue. *Advances in Experimental Medicine and Biology*.

[113] Bruton JD, Place N, Yamada T (2008). Reactive oxygen species and fatigue-induced prolonged low-frequency force depression in skeletal muscle fibres of rats, mice and SOD2 overexpressing mice. *The Journal of Physiology*.

[114] Gülçin I (2006). Antioxidant and antiradical activities of L-carnitine. *Life Sciences*.

[115] Viña J, Gimeno A, Sastre J (2000). Mechanism of free radical production in exhaustive exercise in humans and rats; role of xanthine oxidase and protection by allopurinol. *IUBMB Life*.

[116] Bloomer RJ, Smith WA, Fisher-Wellman KH (2010). Oxidative stress in response to forearm ischemia-reperfusion with and without carnitine administration. *International Journal for Vitamin and Nutrition Research*.

[117] Bloomer RJ, Smith WA (2009). Oxidative stress in response to aerobic and anaerobic power testing: influence of exercise training and carnitine supplementation. *Research in Sports Medicine*.

[118] Casas H, Murtra B, Casas M (2001). Increased blood ammonia in hypoxia during exercise in humans. *Journal of Physiology and Biochemistry*.

[119] Parolin ML, Spriet LL, Hultman E, Hollidge-Horvat MG, Jones NL, Heigenhauser GJF (2000). Regulation of glycogen phosphorylase and PDH during exercise in human skeletal muscle during hypoxia. *American Journal of Physiology: Endocrinology and Metabolism*.

[120] Moretti S, Famularo G, Marcellini S (2002). L-carnitine reduces lymphocyte apoptosis and oxidant stress in HIV-1-infected subjects treated with zidovudine and didanosine. *Antioxidants and Redox Signaling*.

[121] Binienda ZK (2003). Neuroprotective effects of L-carnitine in induced mitochondrial dysfunction. *Annals of the New York Academy of Sciences*.

[122] Kumaran S, Savitha S, Anusuya Devi M, Panneerselvam C (2004). L-carnitine and DL-α-lipoic acid reverse the age-related deficit in glutathione redox state in skeletal muscle and heart tissues. *Mechanisms of Ageing and Development*.

[123] Kumaran S, Subathra M, Balu M, Panneerselvam C (2005). Supplementation of L-carnitine improves mitochondrial enzymes in heart and skeletal muscle of aged rats. *Experimental Aging Research*.

[124] Şener G, Paskaloğlu K, Şatiroglu H, Alican I, Kaçmaz A, Sakarcan A (2004). L-carnitine ameliorates oxidative damage due to chronic renal failure in rats. *Journal of Cardiovascular Pharmacology*.

[125] Virmani A, Gaetani F, Binienda Z, Xu A, Duhart H, Ali SF (2004). Role of mitochondrial dysfunction in neurotoxicity of MPP^+^: partial protection of PC12 cells by acetyl-L-carnitine. *Annals of the New York Academy of Sciences*.

[126] Binienda Z, Przybyla-Zawislak B, Virmani A, Schmued L (2005). L-carnitine and neuroprotection in the animal model of mitochondrial dysfunction. *Annals of the New York Academy of Sciences*.

[127] Keil U, Scherping I, Hauptmann S, Schuessel K, Eckert A, Müller WE (2006). Piracetam improves mitochondrial dysfunction following oxidative stress. *British Journal of Pharmacology*.

[128] Yapar K, Kart A, Karapehlivan M (2007). Hepatoprotective effect of l-carnitine against acute acetaminophen toxicity in mice. *Experimental and Toxicologic Pathology*.

[129] Shen W, Liu K, Tian C (2008). Protective effects of R-α-lipoic acid and acetyl-L-carnitine in MIN6 and isolated rat islet cells chronically exposed to oleic acid. *Journal of Cellular Biochemistry*.

[130] Silva-Adaya D, Pérez-de La Cruz V, Herrera-Mundo MN (2008). Excitotoxic damage, disrupted energy metabolism, and oxidative stress in the rat brain: antioxidant and neuroprotective effects of L-carnitine. *Journal of Neurochemistry*.

[131] Elinos-Calderón D, Robledo-Arratia Y, Pérez-de la Cruz V, Pedraza-Chaverrí J, Ali SF, Santamaría A (2009). Early nerve ending rescue from oxidative damage and energy failure by l-carnitine as post-treatment in two neurotoxic models in rat: Recovery of antioxidant and reductive capacities. *Experimental Brain Research*.

[132] Vamos E, Voros K, Vecsei L, Klivenyi P (2010). Neuroprotective effects of L-carnitine in a transgenic animal model of Huntington's disease. *Biomedicine and Pharmacotherapy*.

[133] Ye J, Li J, Yu Y, Wei Q, Deng W, Yu L (2010). L-carnitine attenuates oxidant injury in HK-2 cells via ROS-mitochondria pathway. *Regulatory Peptides*.

[134] Zhang Z, Zhao M, Wang J, Ding Y, Dai X, Li Y (2010). Effect of acetyl-L-carnitine on the insulin resistance of L6 cells induced by tumor necrosis factor-alpha. *Wei Sheng Yan Jiu*.

[135] Argilés JM, Alvarez B, López-Soriano FJ (1997). The metabolic basis of cancer cachexia. *Medicinal Research Reviews*.

[136] Breitkreutz R, Babylon A, Hack V (2000). Effect of carnitine on muscular glutamate uptake and intramuscular glutathione in malignant diseases. *British Journal of Cancer*.

[137] Ushmorov A, Hack V, Dröge W (1999). Differential reconstitution of mitochondrial respiratory chain activity and plasma redox state by cysteine and ornithine in a model of cancer cachexia. *Cancer Research*.

[138] Hack V, Gross A, Kinscherf R (1996). Abnormal glutathione and sulfate levels after interleukin 6 treatment and in tumor-induced cachexia. *The FASEB Journal*.

[139] Gramignano G, Lusso MR, Madeddu C (2006). Efficacy of l-carnitine administration on fatigue, nutritional status, oxidative stress, and related quality of life in 12 advanced cancer patients undergoing anticancer therapy. *Nutrition*.

[140] Malaguarnera M, Gargante MP, Russo C (2010). L-carnitine supplementation to diet: a new tool in treatment of nonalcoholic steatohepatitisa—a randomized and controlled clinical trial. *The American Journal of Gastroenterology*.

[141] Fatouros IG, Douroudos I, Panagoutsos S (2010). Effects of L-carnitine on oxidative stress responses in patients with renal disease. *Medicine and Science in Sports and Exercise*.

[142] Sitta A, Barschak AG, Deon M (2009). L-carnitine blood levels and oxidative stress in treated phenylketonuric patients. *Cellular and Molecular Neurobiology*.

[143] Mansour HH (2006). Protective role of carnitine ester against radiation-induced oxidative stress in rats. *Pharmacological Research*.

[144] Shaker ME, Houssen ME, Abo-Hashem EM, Ibrahim TM (2009). Comparison of vitamin E, L-carnitine and melatonin in ameliorating carbon tetrachloride and diabetes induced hepatic oxidative stress. *Journal of Physiology and Biochemistry*.

[145] Augustyniak A, Skrzydlewska E (2009). l-Carnitine in the lipid and protein protection against ethanol-induced oxidative stress. *Alcohol*.

[146] Costa RAP, Fernandes MP, De Souza-Pinto NC, Vercesi AE (2013). Protective effects of l-carnitine and piracetam against mitochondrial permeability transition and PC3 cell necrosis induced by simvastatin. *European Journal of Pharmacology*.

[147] DiNicolantonio JJ (2012). CoQ10 and L-carnitine for statin myalgia?. *Expert Review of Cardiovascular Therapy*.

[148] de Lorgeril M, Salen P (2012). New insights into the health effects of dietary saturated and omega-6 and omega-3 polyunsaturated fatty acids. *BMC Medicine*.

[149] Abdukeyum GG, Owen AJ, McLennan PL (2008). Dietary (n-3) long-chain polyunsaturated fatty acids inhibit ischemia and reperfusion arrhythmias and infarction in rat heart not enhanced by ischemic preconditioning. *Journal of Nutrition*.

[150] Zeghichi-Hamri S, de Lorgeril M, Salen P (2010). Protective effect of dietary n-3 polyunsaturated fatty acids on myocardial resistance to ischemia-reperfusion injury in rats. *Nutrition Research*.

[151] Cockbain AJ, Toogood GJ, Hull MA (2012). Omega-3 polyunsaturated fatty acids for the treatment and prevention of colorectal cancer. *Gut*.

[152] Patterson RE, Flatt SW, Newman VA (2011). Marine fatty acid intake is associated with breast cancer prognosis. *Journal of Nutrition*.

[153] Gharekhani A, Khatami MR, Dashti-Khavidaki S (2014). The effect of omega-3 fatty acids on depressive symptoms and inflammatory markers in maintenance hemodialysis patients: a randomized, placebo-controlled clinical trial. *European Journal of Clinical Pharmacology*.

[154] Giles GE, Mahoney CR, Kanarek RB (2013). Omega-3 fatty acids influence mood in healthy and depressed individuale. *Nutrition Reviews*.

[155] Hashimoto K (2006). Glycine transporter inhibitors as therapeutic agents for schizophrenia. *Recent Patents on CNS Drug Discovery*.

[156] Clement AB, Gimpl G, Behl C (2010). Oxidative stress resistance in hippocampal cells is associated with altered membrane fluidity and enhanced nonamyloidogenic cleavage of endogenous amyloid precursor protein. *Free Radical Biology & Medicine*.

[157] Scheuer K (1999). Piracetam improves cognitive performance by restoring neurochemical deficits of the aged rat brain. *Pharmacopsychiatry*.

[158] Schaeffer EL, Gattaz WF (2007). Requirement of hippocampal phospholipase A2 activity for long-term memory retrieval in rats. *Journal of Neural Transmission*.

[159] Carrié I, Abellan Van Kan G, Rolland Y, Gillette-Guyonnet S, Vellas B (2009). PUFA for prevention and treatment of dementia?. *Current Pharmaceutical Design*.

[160] Hooijmans CR, Graven C, Dederen PJ, Tanila H, van Groen T, Kiliaan AJ (2007). Amyloid beta deposition is related to decreased glucose transporter-1 levels and hippocampal atrophy in brains of aged APP/PS1 mice. *Brain Research*.

[161] Lim GP, Calon F, Morihara T (2005). A diet enriched with the omega-3 fatty acid docosahexaenoic acid reduces amyloid burden in an aged Alzheimer mouse model. *Journal of Neuroscience*.

[162] Shinto L, Marracci G, Bumgarner L, Yadav V (2011). The effects of omega-3 fatty acids on matrix metalloproteinase-9 production and cell migration in human immune cells: implications for multiple sclerosis. *Autoimmune Diseases*.

[163] Adamo AM (2014). Nutritional factors and aging in demyelinating diseases. *Genes & Nutrition*.

[164] Fallon EM, Nazarian A, Nehra D, Pan AH, O’Loughlin AA, Puder M (2014). The effect of docosahexaenoic acid on bone microstructure in young mice and bone fracture in neonates. *Journal of Surgical Research*.

[165] Lau BY, Cohen DJ, Ward WE, Ma DW (2013). Investigating the role of polyunsatured fatty acids in bone development using animal models. *Molecules*.

[166] Mangano KM, Sahni S, Kerstetter JE, Kenny AM, Hannan MT (2013). Polyunsaturated fatty acids and their relation with bone and muscle health in adults. *Current Osteoporosis Reports*.

[167] Gingras A, White PJ, Chouinard PY (2007). Long-chain omega-3 fatty acids regulate bovine whole-body protein metabolism by promoting muscle insulin signalling to the Akt-mTOR-S6K1 pathway and insulin sensitivity. *Journal of Physiology*.

[168] Alexander JW, Saito H, Trocki O, Ogle CK (1986). The importance of lipid type in the diet after burn injury. *Annals of Surgery*.

[169] Smith GI, Atherton P, Reeds DN (2011). Dietary omega-3 fatty acid supplementation increases the rate of muscle protein synthesis in older adults: a randomized controlled trial. *The American Journal of Clinical Nutrition*.

[170] Mangano KM, Sahni S, Kerstetter JE, Kenny AM, Hannan MT (2013). Polyunsaturated fatty acids and their relation with bone and muscle health in adults. *Current Osteoporosis Reports*.

[171] Smith GI, Atherton P, Reeds DN (2011). Omega-3 polyunsaturated fatty acids augment the muscle protein anabolic response to hyperinsulinaemia-hyperaminoacidaemia in healthy young and middle-aged men and women. *Clinical Science*.

[172] Liu S, Baracos VE, Quinney HA, Clandinin MT (1994). Dietary *ω*-3 and polyunsaturated fatty acids modify fatty acyl composition and insulin binding in skeletal-muscle sarcolemma. *Biochemical Journal*.

[173] Holness MJ, Smith ND, Greenwood GK, Sugden MC (2004). Acute *ω*-3 fatty acid enrichment selectively reverses high-saturated fat feeding-induced insulin hypersecretion but does not improve peripheral insulin resistance. *Diabetes*.

[174] Borkman M, Chisholm DJ, Furler SM (1989). Effects of fish oil supplementation on glucose and lipid metabolism in NIDDM. *Diabetes*.

[175] Taouis M, Dagou C, Ster C, Durand G, Pinault M, Delarue J (2002). N-3 Polyunsaturated fatty acids prevent the defect of insulin receptor signaling in muscle. *The American Journal of Physiology: Endocrinology and Metabolism*.

[176] Lombardo YB, Chicco AG (2006). Effects of dietary polyunsaturated n-3 fatty acids on dyslipidemia and insulin resistance in rodents and humans. A review. *The Journal of Nutritional Biochemistry*.

[177] Carpentier YA, Portois L, Malaisse WJ (2006). n-3 Fatty acids and the metabolic syndrome. *The American Journal of Clinical Nutrition*.

[178] Fedor D, Kelley DS (2009). Prevention of insulin resistance by n-3 polyunsaturated fatty acids. *Current Opinion in Clinical Nutrition and Metabolic Care*.

[179] Browning LM (2003). n-3 polyunsaturated fatty acids, inflammation and obesity-related disease. *Proceedings of the Nutrition Society*.

[180] Rousseau JH, Kleppinger A, Kenny AM (2009). Self-reported dietary intake of omega-3 fatty acids and association with bone and lower extremity function. *Journal of the American Geriatrics Society*.

[181] Takayama M, Arai Y, Sasaki S (2013). Association of marine-origin N-3 polyunsaturated fatty acids consumption and functional mobility in the community-dwelling oldest old. *The Journal of Nutrition, Health and Aging*.

[182] Hutchins-Wiese HL, Kleppinger A, Annis K (2013). The impact of supplemental N-3 long chain polyunsaturated fatty acids and dietary antioxidants on physical performance in postmenopausal women. *Journal of Nutrition, Health and Aging*.

[183] Rodacki CLN, Rodacki ALF, Pereira G (2012). Fish-oil supplementation enhances the effects of strength training in elderly women. *The American Journal of Clinical Nutrition*.

[184] Maroon J, Bost J (2006). *Fish Oil: The Natural Anti-Inflammatory*.

[185] Calder PC (2006). n-3 Polyunsaturated fatty acids, inflammation, and inflammatory diseases. *The American Journal of Clinical Nutrition*.

[186] Calder PC (1997). n-3 polyunsaturated fatty acids and cytokine production in health and disease. *Annals of Nutrition & Metabolism*.

[187] Billiar TR, Bankey PE, Svingen BA (1988). Fatty acid intake and Kupffer cell function: fish oil alters eicosanoid and monokine production to endotoxin stimulation. *Surgery*.

[188] Hardardottir I, Kinsella JE (1992). Increasing the dietary (n-3) to (n-6) polyunsaturated fatty acid ratio increases tumor necrosis factor production by murine resident peritoneal macrophages without an effect on elicited peritoneal macrophages. *Journal of Nutrition*.

[189] Jolly CA, Jiang Y, Chapkin RS, McMurray DN (1997). Dietary (n-3) polyunsaturated fatty acids suppress murine lymphoproliferation, interleukin-2 secretion, and the formation of diacylglycerol and ceramide. *Journal of Nutrition*.

[190] Endres S, Ghorbani R, Kelley VE (1989). The effect of dietary supplementation with n-3 polyunsaturated fatty acids on the synthesis of interleukin-1 and tumor necrosis factor by mononuclear cells. *The New England Journal of Medicine*.

[191] Bouwens M, van De Rest O, Dellschaft N (2009). Fish-oil supplementation induces antiinflammatory gene expression profiles in human blood mononuclear cells. *The American Journal of Clinical Nutrition*.

[192] Toft AD, Thorn M, Ostrowski K (2000). N-3 polyunsaturated fatty acids do not affect cytokine response to strenuous exercise. *Journal of Applied Physiology*.

[193] Phillips T, Childs AC, Dreon DM, Phinney S, Leeuwenburgh C (2003). A dietary supplement attenuates IL-6 and CRP after eccentric exercise in untrained males. *Medicine and Science in Sports and Exercise*.

[194] Jouris KB, McDaniel JL, Weiss EP (2011). The effect of omega-3 fatty acid supplementation on the inflammatory response to eccentric strength exercise. *Journal of Sports Science and Medicine*.

[195] Ma DWL, Seo J, Davidson LA (2004). n-3 PUFA alter caveolae lipid composition and resident protein localization in mouse colon. *The FASEB Journal*.

[196] Gorjão R, Azevedo-Martins AK, Rodrigues HG (2009). Comparative effects of DHA and EPA on cell function. *Pharmacology and Therapeutics*.

[197] Maxfield FR, Tabas I (2005). Role of cholesterol and lipid organization in disease. *Nature*.

[198] Escribá PV, González-Ros JM, Goñi FM (2008). Membranes: a meeting point for lipids, proteins and therapies. *Journal of Cellular and Molecular Medicine*.

[199] Benabdellah F, Yu H, Brunelle A, Laprévote O, de la Porte S (2009). MALDI reveals membrane lipid profile reversion in MDX mice. *Neurobiology of Disease*.

[200] Masuelli L, Trono P, Marzocchella L (2008). Intercalated disk remodeling in *δ*-sarcoglycan-deficient hamsters fed with an α-linolenic acid-enriched diet. *International Journal of Molecular Medicine*.

[201] Gáti I, Danielsson O, Betmark T, Ernerudh J, Öllinger K, Dizdar N (2007). Effects of inhibitors of the arachidonic acid cascade on primary muscle culture from a Duchenne muscular dystrophy patient. *Prostaglandins Leukotrienes and Essential Fatty Acids*.

[202] Tidball JG (2005). Inflammatory processes in muscle injury and repair. *American Journal of Physiology: Regulatory Integrative and Comparative Physiology*.

[203] Bougnoux P, Hajjaji N, Maheo K, Couet C, Chevalier S (2010). Fatty acids and breast cancer: sensitization to treatments and prevention of metastatic re-growth. *Progress in Lipid Research*.

[204] Ries A, Trottenberg P, Elsner F (2012). A systematic review on the role of fish oil for the treatment of cachexia in advanced cancer: an EPCRC cachexia guidelines project. *Palliative Medicine*.

[205] Fiaccavento R, Carotenuto F, Minieri M (2006). Alpha-linolenic acid-enriched diet prevents myocardial damage and expands longevity in cardiomyopathic hamsters. *The American Journal of Pathology*.

[206] Fiaccavento R, Carotenuto F, Vecchini A (2010). An omega-3 fatty acid-enriched diet prevents skeletal muscle lesions in a hamster model of dystrophy. *The American Journal of Pathology*.

[207] Machado RV, Mauricio AF, Taniguti APT, Ferretti R, Neto HS, Marques MJ (2011). Eicosapentaenoic acid decreases TNF-α and protects dystrophic muscles of mdx mice from degeneration. *Journal of Neuroimmunology*.

[208] Mauricio AF, Minatel E, Neto HS, Marques MJ (2013). Effects of fish oil containing eicosapentaenoic acid and docosahexaenoic acid on dystrophic mdx mice. *Clinical Nutrition*.

[209] Henderson GC, Evans NP, Grange RW, Tuazon MA (2014). Compared with that of MUFA, a high dietary intake of n-3 PUFA does not reduce the degree of pathology in mdx mice. *British Journal of Nutrition*.

[210] Galvao TF, Brown BH, Hecker PA (2012). High intake of saturated fat, but not polyunsaturated fat, improves survival in heart failure despite persistent mitochondrial defects. *Cardiovascular Research*.

[211] Oliver MF, Kurien VA, Greenwood TW (1968). Relation between serum-free-fatty acids and arrhythmias and death after acute myocardial infarction.. *The Lancet*.

[212] Jouven X, Charles MA, Desnos M, Ducimetière P (2001). Circulating nonesterified fatty acid level as a predictive risk factor for sudden death in the population. *Circulation*.

